# Some Spinal Cord Lesions.—IV

**Published:** 1911-08-05

**Authors:** 


### NEUROLOGY.

### SOME SPINAL CORD LESIONS.—IV.

### SYRINGOMYELIA.

Weakness and rigidity of the legs and exaggerated reflexes are rarely the earliest or most prominent indications of this disease, for as a rule they do not occur until the characteristic sensory symptoms have been manifest for some time.

The chief pathological changes in the spinal cord consist of the formation of cavities of varying size, shape, and position, surrounded by neuroglial tissue, in the cervical and upper dorsal regions. The loss of power in the legs and their rigidity are due to a descending degeneration of the pyramidal tracts following compression of these by the primary disease in the upper part of the cord.

The onset is slow and insidious. Loss of sensibility to heat, cold, and pain, tactile sensibility remaining normal, are usually the earliest indications of the disease, followed by atrophy of the muscles of the hands and arms. In a well-marked case the following signs may be found; weakness of the hands and arms; weakness and rigidity of the legs; well-marked fibrillary contractions of the atrophied muscles; weakness of the spinal muscles leading to lateral curvature; tactile sensibility is usually unimpaired; loss of sensation to heat, cold and pain is the most constant and characteristic sign. In some cases perversion of the temperature sensation has been noticed, heating being felt as cold and vice versa. These sensory disturbances are usually confined to the upper half of the body.

The knee-jerks are exaggerated, patellar and ankle clonus may be obtained, and the plantar reflexes are extensor. Atrophy of the muscles of the hands and arms ultimately ensues. The joints may be enlarged as in locomotor ataxy—Charcot's joints. The skin of the fingers may be thin and glossy, and the insensitiveness to pain may cause severe burns to pass unnoticed, with the result that extensive sores and ulcers ensue in some cases. The electrical irritability of the muscles is lowered, and some of the atrophied muscles may give the reaction of degeneration. As a general rule the functions of the bladder and rectum are not impaired. Impairment of the sense of taste and smell, inequality of the pupils, nystagmus, paralysis of one of the vocal cords, the tongue or palate, have all been recorded, but none of these are common. The course of the disease is slow and progressive.

It is most likely to be mistaken for amyotrophic lateral sclerosis on account of the association of spasticity of the legs with atrophy of the muscles of the hands, but the presence of the characteristic sensory phenomena would point to syringomyelia. The gradual and indefinite onset, the absence of bladder and rectum symptoms and of disturbance of the tactile sensibility would distinguish this disease from cervical myelitis.

